# Correlated Imaging of the Equine Hyoid Apparatus Using CT, Micro-CT, and Histology

**DOI:** 10.3389/fvets.2021.652563

**Published:** 2021-07-05

**Authors:** Bettina Hartl, Monika Egerbacher, Sibylle Maria Kneissl

**Affiliations:** ^1^Department of Pathobiology, Institute of Topographic Anatomy, University of Veterinary Medicine Vienna, Vienna, Austria; ^2^Administrative Unit Veterinary Medicine, UMIT Tirol - Private University for The Health Sciences, Medical Informatics and Technology GmbH, Hall in Tirol, Austria; ^3^Department for Small Animals and Horses, Clinical Unit of Diagnostic Imaging, University of Veterinary Medicine Vienna, Vienna, Austria

**Keywords:** equine hyoid apparatus, temporohyoid joint, tympanohyoid, epihyoid, lingual process, joints connecting the hyoid apparatus, mineralization within tympanohyoid

## Abstract

**Background:** Detailed radiological evaluation of the normal hyoid apparatus by computed tomography (CT) has not yet been conducted. Thus, it is unclear what type of junction connects the different parts of the equine hyoid apparatus.

**Objectives:** To describe the normal CT anatomy of the equine hyoid apparatus, and to determine the junction type that connects the different parts of the hyoid apparatus.

**Study Design:** Combination of retrospective study and prospective cadaver study.

**Methods:** The medical records of horses that underwent head CT scans from 2009 to 2018 were retrieved. Inclusion criteria for the CT scans were visibility of at least two of the four junctions of the hyoid apparatus. CT images were analyzed in three different planes. Additionally, 10 cadaver heads were processed using CT, micro-CT of selected joints, and histology of all joints.

**Results:** CT scans of 200 horses fulfilled the inclusion criteria. The tympanohyoid cartilage consisted of hyaline cartilage. Areas of mineralization within the cartilage were visible on CT scans as early as 2 years of age. The epihyoid was not fused with the stylohyoid in one-third of the horses. All horses younger than 2.5 years showed three ossification centers of the basihyoid, and all horses younger than 1.5 years had a non-fullydeveloped lingual process. In total, 10 of 11 horses between 1.5 and 3 years had separate ossification centers of the lingual process. We found a synchondrosis between the styloid process and the stylohyoid bone. The basihyoid and thyrohyoid, as well as the stylohyoid and epiyhoid were connected by a synostosis in two-thirds of the horses. The remaining parts were connected to one another by synovial joints.

**Main limitations:** The junctions studied by histologic examination were from older horses, therefore growing patterns of different bones could not be totally clarified.

**Conclusion:** The temporohyoid joint is a synchondrosis. The epihyoid is an ossification center of the stylohyoid and fuses with the stylohyoid in two-thirds of horses. The lingual process has a separate ossification center.

## Introduction

**Figure 1:**
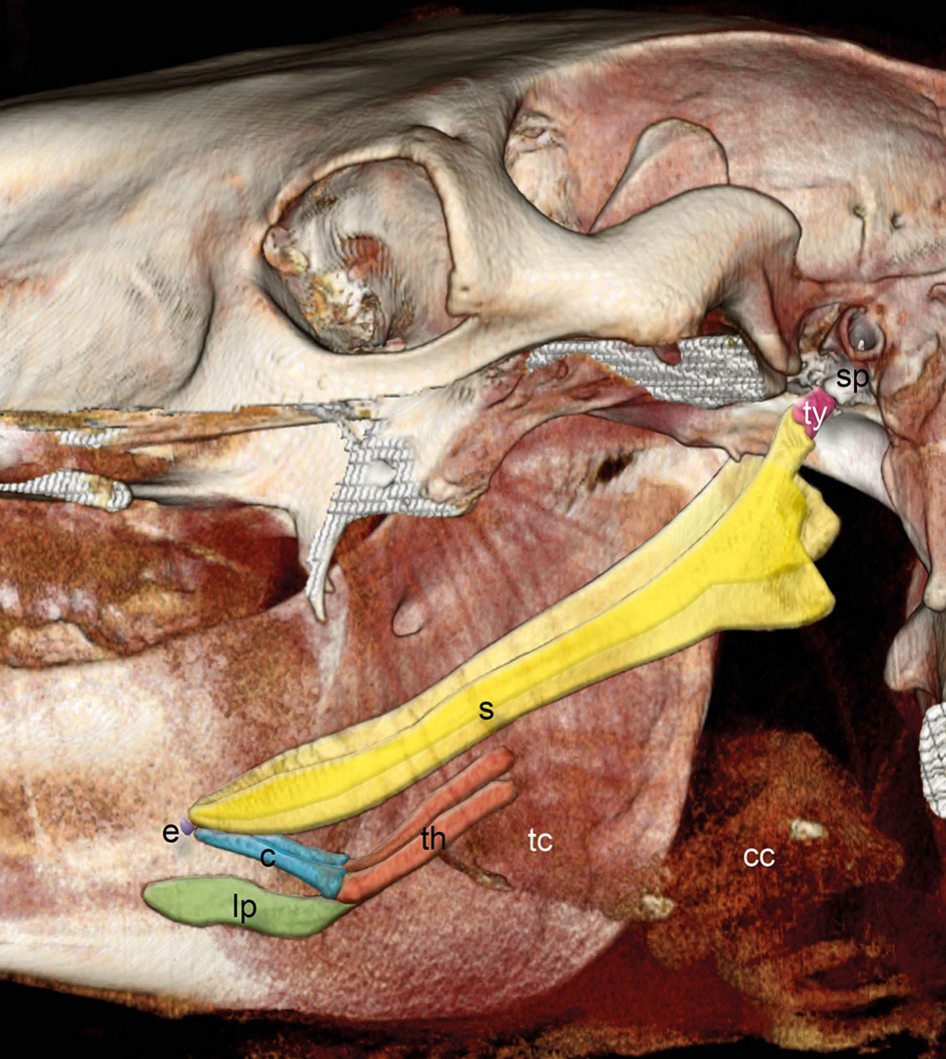
Three-dimensional (3D) reconstruction of an equine skull retrieved from CT images with the left mandible and parts of the left upper jaw removed. Images acquired in the soft tissue algorithm. The styloid process (sp) of the temporal bone is connected via the tympanohyoid (ty) to the stylohyoid bone. Stylohyoid (s), epihyoid (e), ceratohyoid (c), lingual process (lp), and thyrohyoid (th) are indicated. Thyroid cartilage (tc) and cricoid cartilage (cc) could hardly be distinguished.

The hyoid apparatus supports the tongue, pharynx, and larynx and consists of the paired tympanohyoid, stylohyoid, epihyoid, ceratohyoid, unpaired basihyoid, and paired thyrohyoid (Figure 1).

**Figure 2:**
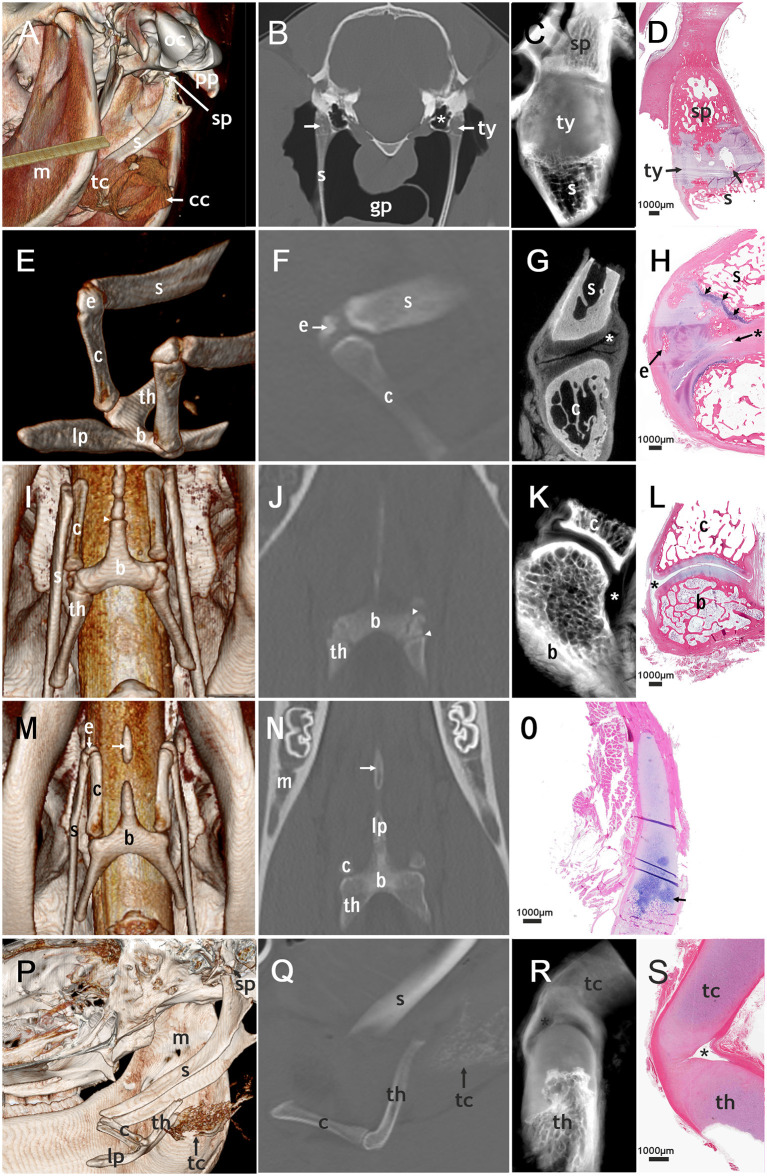
Correlated imaging: first column: 3D image; second column: CT image; third column: micro-CT image; last column: histological section. First row: temporohyoid joint: **(A)**: image from caudoventral. **(B–D)**: transversal plane. **(A)** Occipital condyle (oc) and paracondylar process (pp) are indicated. The tympanohyoid (ty) that connects the stylohyoid process (sp) and the stylohyoid (s) could not be distinguished on this picture. The thyroid cartilage (tc) and cricoid cartilage (cc) surround the tracheal tube. **(B)** areas of mineralization of the tympanohyoid (arrow) in a 17.5-year-old horse. The guttural pouch (gp) surrounds the stylohyoids. **(B,C)**: The tympanic bulla (*) is located directly medial to the temporohyoid joint. **(D)** Mineralization (arrow) in the tympanohyoid cartilage of a 22.1-year-old horse. Second row: joint between stylohyoid (s) and ceratohyoid (c): **(E)**: dorsolateral view, **(F,H)**: sagittal plane, **(G)**: transversal plane. **(E,F,H)**: a separate epihyoid (e) is present. **(H)**: The small joint space (asterisk) lies caudally on this histological section of the 4.8-year-old horse. The epihyoid is surrounded by hyaline cartilage and the epiphyseal plate (arrows) is located between stylohyoid and epihyoid. Ossification is not completed on the proximal end of the ceratohyoid. Third row: joint between ceratohyoid (c) and basihyoid (b): **(I)**: ventral view, **(J)**: coronary plane, **(K,L)**: sagittal plane. **(I,J)**: The epiphyseal plates (arrowheads) of the basihyoid are visible. **(K,L)**: The spacious joint space is marked with an asterisk. Fourth row: lingual process: **(M)**: ventral view **(N)**: coronary plane, no micro-CT-image of the lingual process **(O)**: sagittal plane. **(M,N)**: The separate ossification center of the lingual process (arrow) is visible. **(O)**: The epiphyseal plate of the basihyoid of a 16-days-old foal is marked with an arrow. Fifth row: thyrohyoid joint: **(P)** lateral view, slightly from ventral **(Q–S)**: sagittal plane. **(P,Q)**: The thyrohyoid cartilage (tc) can be visualized to some extent. **(R,S)**: The joint space (asterisk) is small and can only be seen on micro-CT images and histological slides. Histological images: H&E, magnification x20.

The temporohyoid joint connects the stylohyoid to the petrous part of the temporal bone via the tympanohyoid (Figure 2A), which consists of fibrocartilage in horses and cows (1–5). The equine epihyoid is a lentil-shaped bone that fuses with the stylohyoid at an early stage (1, 5–7). In pigs, the ligamentum epihyoideum represents the epihyoid (1, 5, 8), whereas in humans, the tympanohyoid, stylohyoid and epihyoid are replaced by the ligamentum stylohyoideum (5, 9). The epihyoid of cows and dogs is connected to the stylohyoid and ceratohyoid by a synovial joint (1, 5, 8). In the domestic cat, the epihyoid is connected to the stylohyoid and ceratohyoid by hyaline cartilage synchondrosis (10). Some studies have shown that horses have synovial joints between the stylohyoid and epihyoid and between the epihyoid and ceratohyoid, similar to cows (1, 3, 8, 11). However, others have found a synchondrosis between the same bones (6, 7, 12, 13). Moreover, one study documented only one synovial joint between the stylohyoid and ceratohyoid (14). The ceratohyoid is connected to the basihyoid by a synovial joint (1–3, 5–8, 12).

The basihyoid consists of the corpus embedded within the root of the tongue and the lingual process, which lies within the body of the tongue. The paired thyrohyoids are connected to the basihyoid by a synostosis (1, 5–8, 12); however, this synostosis develops only in older horses (15). The thyrohyoid is connected to the thyroid cartilage of the larynx through a synovial joint (Figures 1, 2P–S) (5, 7, 8).

The complex anatomy of the equine head and superimposition of multiple structures make it difficult to interpret equine skull radiographs (16–19), and evaluation of the hyoid apparatus using guttural pouch endoscopy is limited to the proximal stylohyoid and temporohyoid joint (18–20). Thus, computed tomography (CT) is the imaging modality of choice for visualizing the structures of the equine head (20, 21). Ultrasonographic examination is restricted to the ventrally located parts, including basihyoid and lingual process (22).

There have been few reports on the normal CT anatomy of the hyoid apparatus. Two authors (16, 23) published labeled transverse CT images of adult horses, however these images did not depict the epihyoid and junctions connecting the different parts of the hyoid apparatus. Smallwood et al. (24) published transverse CT images for foals but the study had labeling inconsistencies, as the epihyoid was marked on a transverse CT image.

Therefore, the goal of this study was to use CT and micro-CT to illustrate the normal anatomy of the complete hyoid apparatus, including the epihyoid and all junctions. Furthermore, histology was used to show the type of junctions that interconnect the different parts of the hyoid apparatus.

## Materials and Methods

### Medical Record Review

The medical records of horses that underwent a head CT scan between 2009 and 2018 were reviewed. Inclusion criteria for the CT scans were visibility of at least two of the four joints of the hyoid apparatus. Information retrieved from the medical records included age, breed, sex, use, and CT diagnosis.

### Cadaver Study

We processed 10 cadaver heads of horses of various breeds from the anatomical collection of the University of Veterinary Medicine (Vienna, Austria). The horses were euthanized in the Equine Clinic of the university, for reasons unrelated to disease of the head. CT scans of the heads were performed within 54 h after euthanization, from rostral of the lingual process to caudal to the tympanic bullae. The heads were positioned standing on their mandibles.

After the CT scan, the hyoid apparatus was isolated, therefor the lingual process was palpated in the intermandibular region and separated from the surrounding musculature. The temporohyoid joint was visualized approaching it from a caudal opening in the guttural pouch. The hyoid apparatus was disconnected from the skull proximally to the temporohyoid joint using the Satterlee amputation saw (Medicon eG, Tuttlingen, Germany). After the muscles were removed, the joints were harvested using the amputation saw and immediately fixed in 4% neutral buffered formalin.

When changes in a joint were detected on CT scans, we performed micro-CT of that joint. One set of unaltered joints (temporohyoid joint, styloceratohyoid/epiceratohyoid joint, ceratobasihyoid joint and thyrohyoid joint) was also subjected to micro-CT. Hyoid specimens were mounted in plastic containers containing 70% ethanol and scanned using the Scanco μCT35 desktop microCT scanner (Scanco Medical AG, Brüttisellen, Switzerland) at 70 kVp peak voltage and 114 μA current. The emitted X-ray spectrum was filtered using a 0.5 mm aluminum filter. Projection images were acquired at 800 ms exposure time per projection and an angular increment of 0.36° between projections. Isotropic voxel resolution in the reconstructed image volumes was 30.0 μm. Reconstructed tomographic slices were exported as a sequence of Dicom images.

All isolated joints of the hyoid apparatus and two lingual processes were prepared for histological examination. They were fixed in 4% neutral buffered formalin for at least 1 day. Then specimens were cut either sagittally or transversally using a diamond band saw (cut-grinder charly, Patho-Service GmbH, Oststeinbek, Germany). The cut-direction was sagittal on the left side and transversal on the right side. Subsequently, specimens were further fixed in 4% neutral buffered formalin for at least 3 days. Thereafter, specimens were decalcified either in 50% formic acid for 21–148 days or in 8% EDTA for 148-226 days. The decalcified specimens were embedded in paraffin wax, sectioned at 4 μm, and stained with hematoxylin and eosin. Micrographs of the histological sections were taken using a slide scanner at a low-power objective (20x) (Aperio ScanScope® CS, Leica Biosystems, Bern, Switzerland).

### CT Analysis

CT was performed using the SOMATOM® Emotion 16c scanner (Siemens Healthcare, Erlangen, Germany). The technical parameters were as follows: axial type of acquisition, 0.8 mm collimation thickness, 0.5 mm image reconstruction interval, 0.5 s/rotation, 130 kV [peak], and 160-180 mA. The field of view was 16.6 × 16.6 cm and the matrix was 512 × 512. The scans were reconstructed using a soft tissue and bone algorithm.

Images were reviewed on a Dicom workstation (eFilm Workstation 4.2; Merge Healthcare Inc, Chicago, IL, USA). Images were analyzed independently by an equine veterinary surgeon (BH) and discussed with a trained and experienced veterinary radiologist (SK). All images were evaluated twice in three orthogonal planes (transverse, dorsal, sagittal), on independent occasions. For each horse a CT scoring sheet was used, in which patient identity number and name, date of review and CT scan was listed. Furthermore, it was documented whether all structures were analyzable. When bony changes that were not in the proximity of the joint, such as enlargement of the bone, evidence of fracture, lytic osseous change, absence of a bone, deformities or sclerosis were present, the bone was excluded. Joints that showed abnormal radiological changes such as the presence of osteophytes, irregularity of the joint surface or narrowing of the tympanohyoid, were excluded.

### Statistical Analysis

Logistic regression analysis was used to evaluate the possible association between age and mineralization in the tympanohyoid cartilage, and an association between age and the presence of separate epihyoids. The chi-square test was performed to assess the association of mineralization within the tympanohyoid and the presence of a separate epiphyoid for each side.

## Results

### Horses Sampled

In total, 200 horses fulfilled the inclusion criteria including 113 males, 82 females, and 5 horses with no gender record. The age of the horses ranged from 16 days to 29.6 years (mean age, 12.9 years).

### Cadaver Heads

CT scans and dissection could be performed within 24 h after euthanasia in six cadvaver heads, within 48 h in two heads and within 54 h in one head. In another head, the CT scan was conducted within 24 h after euthanasia, afterwards the cadaver head was frozen and stored at−20°C and dissected after two and a half weeks.

### Temporohyoid Joint (Articulatio Temporohyoidea)

**Figure 3:**
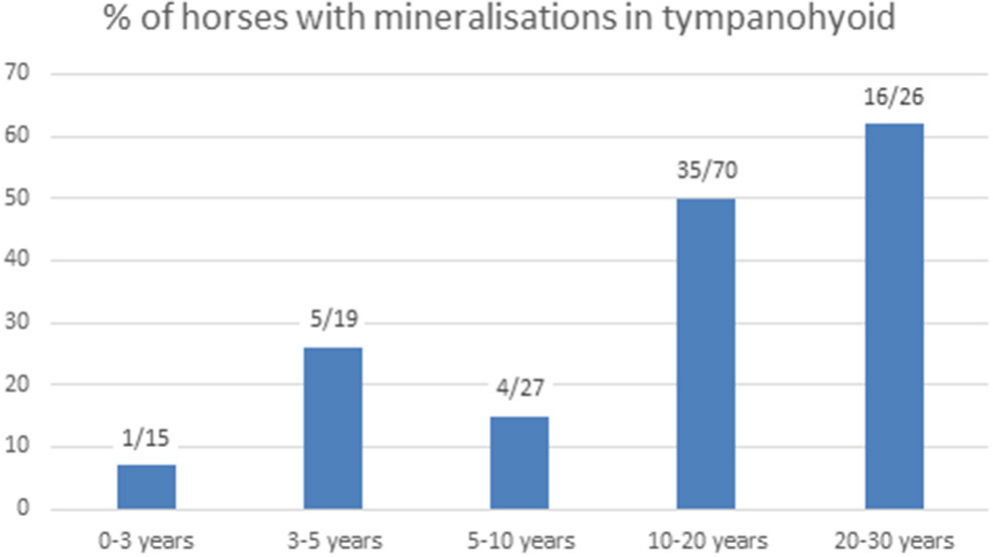
Number of horses with mineralization in the tympanohyoid and percentage in the different age groups.

We were able to evaluate both temporohyoid joints in 159 of the 200 horses, and in two additional horses only one temporohyoid joint was visible on the CT scans. Mineralization within the tympanohyoid cartilage was visible on the CT scans in 38.4% (61/159) of horses (Figures 2B,D). In 34.6% (55/159) and 3.8% (6/159) of horses, mineralization occurred bilaterally and unilaterally, respectively. The mean age of horses showing mineralization was 15.6 years (range, 2.3-29.6 years). Logistic regression revealed no association between age and mineralization within the tympanohyoid; however, mineralization was very rare in horses between 0 and 3 years of age and was more frequent amongst older horses (Figure 3). Mineralization within the tympanohyoid was not associated with the presence of a separate epihyoid on the ipsilateral side.

Histological sections provided evidence that the tympanohyoid cartilage consisted of hyaline cartilage (Figure 2D). Ossification between tympanohyoid and stylohyoid was not completed in examined horses up to 4.8 years old. In older horses, a synchondrosis united the two structures. The styloid process represented as a small cylinder filled with cancellous bone, the distal end of which appeared to be frayed. Hyaline cartilage extended into these gaps. The distal blunt ending was covered with a thin layer of mineralized cartilage, with occasional areas of degenerated tissue in all horses over 1 month old. The styloid process was coated with fibrocartilage.

Two temporohyoid joints from the left side of two different horses were examined by micro-CT. Due to changes in one temporohyoid joint, we could only use the micro-CT images of one temporohyoid joint to describe the normal anatomy. On micro-CT images the styloid process was concave at the distal end (Figure 2C). The transition between styloid process and tympanohyoid and between tympanohyoid and stylohyoid appeared to be interlocking. The stylohyoid was concave at the proximal end.

### Styloepihyoid Joint (Articulatio Styloepihyoidea—Joint Between the Stylohyoid and Epihyoid)

**Figure 4:**
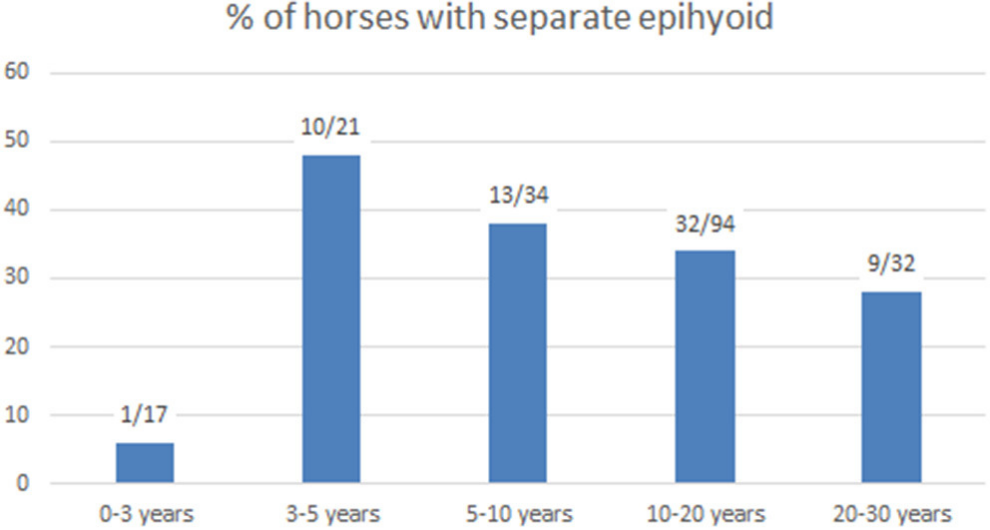
Number of horses with separate epihyoids and percentage in different age groups.

We examined the connection between the stylohyoid and epihyoid on all CT scans. A separate epihyoid was present in 33% (66/200) of horses (Figures 2E,F). In 25% (49/200) of horses, the epihyoid could be identified bilaterally and in 9% (17/200), it was only present on one side. The epihyoid was best visualized on sagittal thick-slice MPRs or in a dorsal plane. It was difficult to identify the epihyoid on transverse CT images. Logistic regression revealed no association between age and the presence of a separate epihyoid. When analyzed by age group, a separate epihyoid was found to be very rare in horses under 3 years of age. Whereas, it was common between 3 and 5 years and decreased with increasing age (Figure 4).

In horses with a separate epihyoid, a joint between the stylohyoid and epihyoid was present on the CT images. However, on histological sections and in micro-CT images there was no evidence of a synovial joint between the stylohyoid and epihyoid but the two bones were connected by hyaline cartilage. In juvenile horses a growth plate was visible between the stylohyoid and epihyoid (Figure 2H).

None of the styloceratohyoid/epiceratohyoid joints that were examined by micro-CT, had a separate epihyoid, hence we were not able to evaluate the styloepihyoid joint by micro-CT.

### Styloceratohyoid/Epiceratohyoid Joint (Articulatio Styloceratohyoidea/Epiceratohyoidea—Joint Between the Stylohyoid/Epihyoid and Ceratohyoid)

We assessed the joint between the epihyoid and ceratohyoid (or in cases of a fused epihyoid, the joint between the stylohyoid and ceratohyoid) in all horses.

In histological sections, we identified a synovial capsule and hyaline cartilage covering the adjacent bones of this joint. In some sections, a synovial fold, as well as a recess of the synovial capsule containing synovial villi, occurred caudally. On the rostral aspect of the joint, ligaments bypassed the interarticular space. When the epihyoid was not fused with the stylohyoid, a joint space was visible between the epihyoid and ceratohyoid but not between the stylohyoid and epihyoid. In this case, cartilage connected the stylohyoid and epihyoid. In foals, a growth plate between the stylohyoid was present. In one horse (4.8 years old) ossification zones in the epihyoid and an epiphyseal plate of the stylohyoid were observed.

In three styloceratohyoid/epicertohyoid joints from the left side of three different horses micro-CT scanning was performed. Due to changes in two styloceratohyoid/epiceratohyoid joints, we could only use the micro-CT images of one styloceratohyoid/epiceratohyoid joint to describe the normal anatomy. On micro-CT images, the narrow joint space (Figure 2G) became more spacious when going caudally on transverse slides. The cartilage covering the adjacent bones appeared thickest on transverse images, halfway of the rostrocaudal extent of the articular surface of the ceratohyoid.

### Ceratobasihyoid Joint (Articulatio Ceratobasihyoidea—Joint Between the Ceratohyoid and Basihyoid)

The ceratobasihyoid joint was visible on all CT scans. In histological sections, we discovered a synovial capsule and hyaline cartilage on the adjacent bones (Figure 2L). In some samples, we identified a synovial fold cranially and a fat pad on the caudal part of the joint located underneath the articular ligaments.

Two ceratobasihyoid joints from the left side of two different horses were evaluated using micro-CT. Due to changes in one ceratobasihyoid joint, we could only use the micro-CT images of one ceratobasihyoid joint to describe the normal anatomy. The joint showed a spacious joint space (Figure 2K), with a larger recess on the caudolateral aspect and a smaller recess on the craniomedial side.

### Basihyoid Including the Lingual Process and Thyrohyoid

**Figure 5:**
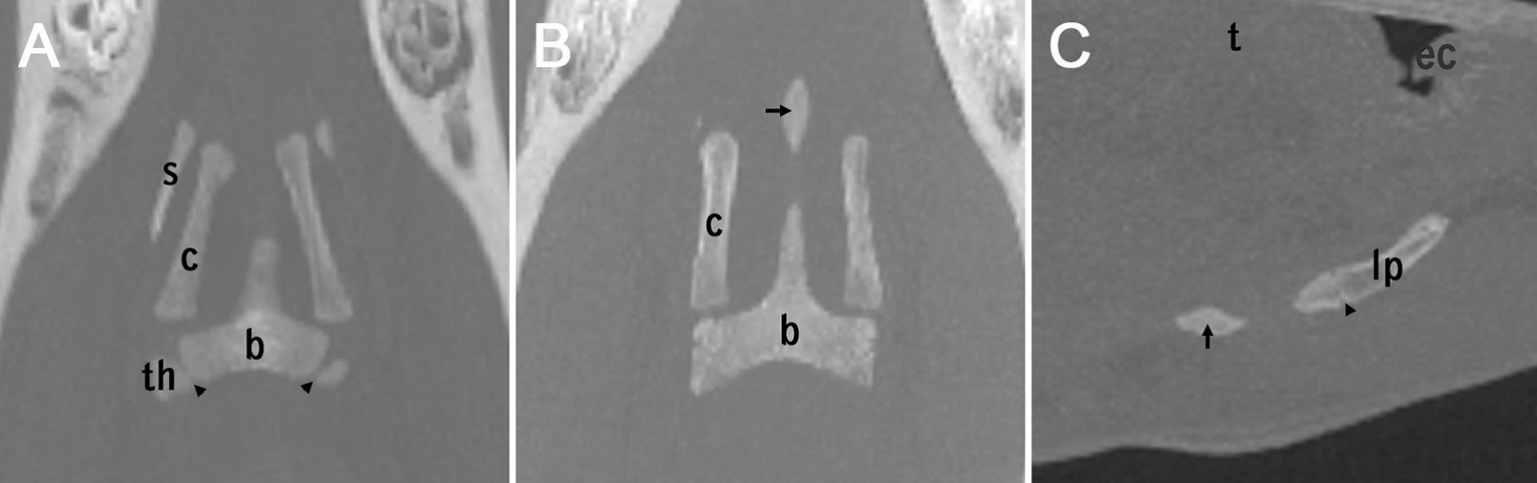
Three different appearances of the lingual process **(A,B)**: coronary plane **(C)**: sagittal plane. **(A)**: non-fully developed lingual process in a 1-year-old horse. The epiphyseal plates of the basihyoid are marked by arrowheads. **(B)** A separate ossification center of the lingual process (arrow) in a 2-year-old horse. **(C)** A separate ossification center of the lingual process (arrow) and an growth plate of the lingual process (arrowhead) between the second ossification center of the lingual process and the basihyoid, in a 2-year-old horse. The epiglottis is indicated with ec (epiglottic cartilage).

The basihyoid was visible on all CT scans. CT evaluation revealed three ossification centers of the basihyoid, one for the corpus and one each for every thyrohyoid in all horses under 2.5 years of age (Figures 2I,J, 5A). The mean age of horses with three ossification centers of the basihyoid was 1.6 years (range, 0.1–3.4 years), and one horse was 3.4 years old.

The lingual process of the basihyoid was visible on all CT scans, but its appearance differed depending on age (Figures 2I,J,M,N,P, 5A–C). All horses under 1.5 years and one 2.4 year-old horse had a non-fully developed lingual process that was approximately half the size of a full-grown lingual process (Figures 2O, 5A). Eight of nine horses with this non-fully developed lingual process also showed three ossification centers of the basihyoid.

In 14 horses ranging in age from 1.8 to 6 years, a separate ossification center of the lingual process was visible on CT scans (Figures 2M,N, 5B,C). Ninety percent (10/11) of horses between 1.5 and 3 years old had this separate ossification center of the lingual process. One horse (2.4 years) in this age group did not have a separate ossification center and had a non-fully developed lingual process. Sixty-seven percent (4/6) of horses between 1.9 and 2.1 years old had an intercalated ossification center between the corpus and the ossification center of the apophysis of the lingual process (Figure 5C).

### Basithyrohyoid Joint (Articulatio Basithyrohyoidea—Joint Between the Basihyoid and Thyrohyoid)

The connection between the basihyoid and thyrohyoid bones was visible on all CT scans. The basihyoid and thyrohyoid were not fused in horses under 2.5 years of age. In horses older than 2.5 years, a synostosis united the two bones on either side.

On histological sections, ossification was not completed in horses under 4.8 years of age; however in horses older than 4.8 years, the two bones could no longer be distinguished from one another. This connection was not examined using micro-CT scanning.

### Thyrohyoid Joint (Articulatio Thyrohyoidea—Joint Between the Thyrohyoid and Rostral Cornu of the Thyroid Cartilage)

On CT scans, the thyrohyoid joint could not be visualized (Figure 2Q). Histological and micro-CT images showed that this joint had hyaline cartilage on both adjacent bones and a very small joint capsule in histological sections and micro-CT-images (Figures 2R,S). One thyrohyoid joint of the left side was analyzed using micro-CT. The transition between bone and cartilage of the thyrohyoid appeared to be frayed on micro-CT images. The joint capsule was clearly visible and surrounded a small joint cavity.

## Discussion

The aim of this study was to describe the normal CT anatomy of the equine hyoid apparatus. Furthermore, we aimed to clarify which type of junction connects the different parts of the hyoid apparatus.

Junctions can be classified into three groups: fibrous junctions, cartilaginous junctions and synovial junctions. Cartilaginous junctions can be further subclassified into synchondroses, in which contiguous skeletal structures are united by hyaline cartilage and symphyses where two structures are united by fibrocartilage.

The tympanohyoid connects the stylohyoid to the styloid process of the temporal bone and reportedly consists of fibrocartilage (1–5). In our study, the tympanohyoid was found to consist of hyaline cartilage. The connection between the styloid process and the stylohyoid represents a synchondrosis (Table 1) similar to the connection between the rib bone and rib cartilage in adult animals, as described by Witter et al. (25).

**Table 1:** Classification of connection between the different parts of the hyoid apparatus.

**United bones**	**Connection**	**Type of connection**
*Styloid process*- *stylohyoid*	Temporohyoid joint	Synchondrosis
*Stylohyoid - epihyoid*	Styloepihyoid connection	Synostosis
*Stylohyoid/epihyoid - ceratohyoid*	Styloceratohyoid/epiceratohyoid joint	Synovial joint
*Ceratohyoid - basihyoid*	Ceratobasihyoid joint	Synovial joint
*Basihyoid - thyrohyoid*	Basithyrohyoid connection	Synostosis
*Thyrohyoid - thyroid cartilage*	Thyrohyoid joint	Synovial joint

In a previous study, mineralization within the tympanohyoid cartilage was linked to increasing age and became evident in conjunction with remodeling of the proximal stylohyoid bone (4). In this study, we found these mineralized areas in approximately one-third of the horses. Mineralization was associated with age, as it was rare in horses younger than 3 years old, but occurred in approximately two-thirds of horses between 20 and 30 years old. We could only differentiate between calcification and ossification on histological sections but not on normal CT scans. We suggest that this mineralization occurs similar to calcifications in the costal cartilage of adult animals, where cartilage is preserved in the area of transition (25). Comparison of micro-CT imaging and histology was difficult due to the low number of joints subjected to micro-CT. However, histological findings confirmed the micro-CT images regarding the presence of a synovial joint. Histology was superior for assessing growth plates and cellular structures.

Varying opinions exist concerning the epihyoid. Some authors describe it as a small bone that fuses with the stylohyoid at an early stage (5, 6), but it has also been suggested that the epihyoid is an ossification center between the stylohyoid and ceratohyoid, which often does not develop to a discrete bone (12).

In this study, a separate epihyoid was visible on CT scans in one-third of the horses. On CT images, we found separate epihyoids in only one of 17 horses under 3 years of age, in accordance with the findings by Franck (2), which showed that the epihyoid is still cartilaginous in the third year. In horses older than 3 years of age, a separate epihyoid was frequently observed on CT scans, with a 48% incidence in horses 3–5 years old. This percentage decreased with age, supporting Leyh's conclusion that the epihyoid fuses with the stylohyoid in older horses (3).

Smallwood et al. (24) labeled the epihyoid on a transverse CT image. We found that the epihyoid was best visualized on sagittal thick-slice MPRs or in a dorsal plane. We found it very difficult to identify the epihyoid on a transverse section. It is possible that the hyoid apparatus of the foal in Smallwood's paper had an uncommon angulation of the different parts of the hyoid apparatus.

On histological sections, the epihyoid was connected to the stylohyoid. Therefore, we presume that the epihyoid is an ossification center of the stylohyoid.

The styloepihyoid joint was termed a synchondrosis by Schmaltz (6) and a synovial joint by others (2, 3). In our study, a cartilaginous connection existed where the epihyoid was not fused with the stylohyoid on the CT scans. We did not detect a synovial joint between the epihyoid and stylohyoid in the histological sections. The styloceratohyoid/epiceratohyoid joint was described as a synchondrosis by Schmaltz (6) and a synovial joint by others (3, 11). In our study, this junction showed several characteristics of a synovial joint, such as a synovial capsule, hyaline cartilage covering the adjacent bones, a synovial fold, and a fat pad.

We, therefore, conclude that there is only one joint space between the ceratohyoid and stylohyoid/epihyoid.

In the literature, the junction between the ceratohyoid and basihyoid is described as a synovial joint with a joint capsule, capsular ligaments, an articular cavity and a convex articular surface of the basihyoid (2, 3, 14). Our study confirmed these findings.

The basihyoid reportedly consists of three ossification centers in young horses that merge later on (2, 12, 14). Of these three ossification centers, two form the thyrohyoids and one develops into the corpus, including the lingual process (2, 12). We also found these three ossification centers in horses younger than 2.4 years old. Additionally, we discovered a separate ossification center for the lingual process in horses between 1.5 and 3 years old. In horses under 1.5 years of age, we observed a non-fully developed lingual process, and in horses about 2 years old, an intercalated ossification center between corpus and ossification center of the apophysis of the lingual process was common.

We conclude that findings previously applied to the ossification centers of the basihyoid, can in fact only be correctly applied to horses <2.4 years old. In contrast to current anatomical doctrine (5), but in accordance with older anatomical textbooks, that termed the thyrohyoids laryngeal horns (6), laryngeal cornua (2) or fork processes (3) of the basihyoid, we now consider the thyrohyoids as ossification centers of the basihyoid rather than a separate bone. Therefore, the thyrohyoids should rather be named thyrohyoid processes.

The lingual process is not-fully developed in horses <1.5 years old. Between 1.5 and 3 years, a separate ossification center for the lingual process develops (Table 2).

**Table 2:** Form of appearance, time frame of their visibility, and detection method of this appearance of the different components of the hyoid apparatus.

**Structure**	**Form of appearance**	**Time frame of visibility**	**Detection method**
*Tympanohyoid*	Ossification not completed between the tympanohyoid and stylohyoid	16 days−4.8 years	Histology
*Stylohyoid*	Growthplate between stylohyoid and epihyoid	16 days−4.8 years	Histology
*Epihyoid*	Cartilaginous	<3 years	Histology
	Ossified	3-5 years	CT, Histology
	Fused with the stylohyoid	>5 years	CT, Histology
*Ceratohyoid*	Ossification not completed proximally	16 days−4.8 years	Histology
*Basihyoid*	Rudimental lingual process	<1.5 years	CT
	Separate ossification center of lp	1.8-6 years	CT
	Thyrohyoid non-fused with basihyoid	<2.5 years	CT

The thyrohyoid directs caudally and has a cartilaginous ending. The thyrohyoid joint is reportedly a synovial joint (5, 8), which was confirmed in our study. Due to the cartilaginous endings, we were not able to visualize this joint on normal CT scans.

## Conclusions

This study clarified which joints connect the different parts of the equine hyoid apparatus and described the normal anatomy on CT scans. The epihyoid fused with the stylohyoid in two-thirds of the horses and appeared to be an ossification center of the stylohyoid. We found that the thyrohyoids are ossification centers of the basihyoid, as is the lingual process. Therefore, the equine thyrohyoids should rather be renamed thyroid processes.

A detailed description and prevalence of changes in the entire hyoid apparatus are of clinical relevance. To date, research has mainly focused on the temporohyoid joint; however, other joints likely have an impact on the development of temporohyoid osteoarthropathy. Potentially, the fusion or non-fusion of the epihyoid with the stylohyoid could influence the development of THO. Tongue manipulation eventually provokes alterations of the hyoid apparatus. These issues should be further addressed in future research projects.

## Data Availability Statement

The original contributions presented in the study are included in the article/supplementary material, further inquiries can be directed to the corresponding author/s.

## Ethics Statement

Ethical review and approval was not required for the animal study because of national legislation. All investigated premortem computed tomographic data were obtained for clinical reasons non-related to this study. Written informed consent was obtained from the owners for the participation of their animals in this study.

## Author Contributions

BH was the main author of the manuscript, performed the dissection, histologic evaluation, and retrospective evaluation of the CT images. ME assisted with manuscript preparation and supervised the histologic evaluation. SK obtained the figures, assisted in CT image evaluation, and manuscript preparation. All authors gave their final approval of the manuscript.

## Conflict of Interest

The authors declare that the research was conducted in the absence of any commercial or financial relationships that could be construed as a potential conflict of interest.
